# Supplementary material for: Inflammatory Bowel Disease: Are Symptoms and Diet Linked?
Source: Nutrients. 2020 Sep 29;12(10):2975. doi: 10.3390/nu12102975 (PMC7650696; doi:10.3390/nu12102975)
Supplement: Supplementary file 1 [file nutrients-12-02975-s001.zip › Supplementary/HMorton Supplementary Tables S1 S2 S3.docx]

**Supplementary Table S1.** Dietary Elements most frequently identified in IBD Symptom Onset, according to disease subtype.

| **IBD**  *n*=165 |  | **CD**  *n*=146 |  | **UC**  *n*=75 |  |
| --- | --- | --- | --- | --- | --- |
| Deep Fried^1^ | 73% | Deep Fried^1^ | 74% | Deep Fried^1^ | 67% |
| Apple | 63% | Apple | 66% | Alcohol – any | 64% |
| Full-grain bread | 62% | Full-grain bread | 66% | Apple | 56% |
| Ice-cream | 61% | Ice-cream | 63% | Cabbage | 56% |
| Wheat | 59% | Chilli sauce | 63% | Wheat | 56% |
| Kiwifruit | 59% | Kiwifruit | 61% | Ice-cream | 56% |
| Corn | 59% | Wholemeal bread | 61% | Coffee | 56% |
| Cabbage | 57% | Chilli | 59% | Kiwifruit | 53% |
| Onion | 57% | Onion | 59% | Broccoli | 53% |
| Fried^1^ | 57% | Tomato | 59% | Corn | 53% |
| Chilli | 56% | Wheat | 59% | White bread | 53% |
| Chilli sauce | 56% | Corn | 58% | Muesli | 53% |
| Coffee | 56% | Bran based cereal | 58% | Cow’s Milk – light | 53% |
| Fruit juice | 56% | Fruit juice | 58% | Beer | 53% |
| Broccoli | 55% | Fried^1^ | 58% | Fried^1^ | 53% |
| Wholemeal bread | 55% | Cabbage | 57% | Full-grain bread | 51% |
| Bran based cereal | 55% | Brown bread | 57% | Cream | 51% |
| Muesli | 55% | Cheese – hard | 57% | Chilli | 49% |
| Cream | 55% | Cream | 57% | Garlic | 49% |
| Wheat based cereal | 55% | Broccoli | 55% | Onion | 49% |
| Alcohol – any | 55% | Wheat based cereal | 55% | Wheat based cereal | 49% |
| Wine | 55% | Yoghurt – fruit | 55% | Wine | 49% |
| Brown bread | 54% | Chocolate | 55% | Fruit juice | 49% |
| White bread | 54% | Coffee | 55% | Chocolate | 49% |
| Chocolate | 54% | Wine | 55% | Cow’s Milk – whole | 49% |

Cooking methods are marked with ^1^.

**Supplementary Table S2.** Dietary Elements most frequently identified in IBD Symptom Exacerbation, according to disease subtype.

| **IBD**  *n*=165 |  | **CD**  *n*=146 |  | **UC**  *n*=75 |  |
| --- | --- | --- | --- | --- | --- |
| Deep Fried^1^ | 60% | Deep Fried^1^ | 57% | Deep Fried^1^ | 67% |
| Full-grain bread | 55% | Full-grain bread | 51% | Alcohol – any | 65% |
| Alcohol – any | 49% | Chilli sauce | 49% | Full-grain bread | 63% |
| Cabbage | 48% | Cabbage | 45% | Cabbage | 57% |
| Chilli sauce | 48% | Onion | 44% | Wholemeal bread | 57% |
| Ice-cream | 47% | Chilli | 43% | Ice-cream | 57% |
| Wheat | 46% | Wheat | 43% | Cow’s Milk – whole | 57% |
| Wholemeal bread | 46% | Ice-cream | 43% | Muesli | 55% |
| Muesli | 46% | Alcohol – any | 43% | Wine - red or white | 55% |
| Chilli | 45% | Muesli | 42% | Wheat | 53% |
| Onion | 45% | Apple | 41% | Chilli | 51% |
| Cow’s Milk – whole | 45% | Corn | 41% | Popcorn | 51% |
| Fried^1^ | 44% | Peanut | 41% | Wheat based cereal | 51% |
| Broccoli | 43% | Wholemeal bread | 41% | Coffee | 51% |
| Cream | 43% | Fried^1^ | 41% | Fried^1^ | 51% |
| Coffee | 43% | Broccoli | 40% | Broccoli | 49% |
| Apple | 42% | Cream | 40% | Cream | 49% |
| Popcorn | 42% | Orange | 39% | Chilli sauce | 49% |
| Wheat based cereal | 42% | Tomato | 39% | Muesli bars | 49% |
| Corn | 41% | Cashew nuts | 39% | Pizza | 49% |
| Wine | 41% | Cow’s Milk – whole | 38% | Apple | 47% |
| Brown bread | 40% | Fruit juice | 38% | Beer | 47% |
| Peanut | 40% | Garlic | 38% | Onion | 47% |
| Fruit juice | 40% | Kiwifruit | 38% | White bread | 47% |
| Garlic | 40% | Brown bread | 38% | Cow’s Milk – light | 47% |

Cooking methods are marked with ^1^.

**Supplementary Table S3.** Dietary Elements most frequently identified in IBD Symptom Reduction, according to disease subtype.

| **IBD**  *n*=165 |  | **CD**  *n*=146 |  | **UC**  *n*=75 |  |
| --- | --- | --- | --- | --- | --- |
| Banana | 39% | Banana | 37% | Chicken | 50% |
| Rice | 35% | White bread | 33% | Rice | 50% |
| White bread | 33% | Rice | 25% | Fish – non oily | 46% |
| Chicken | 32% | Kumara | 23% | Fish – oily | 46% |
| Fish – oily | 28% | Eggs | 21% | Banana | 42% |
| Fish – non oily | 27% | Chicken | 21% | Rice based cereal | 42% |
| Eggs | 26% | Yoghurt – fruit | 19% | Baked^1^ | 38% |
| Pumpkin | 24% | Fish – oily | 19% | Pumpkin | 33% |
| Yoghurt – fruit | 24% | Marmite/Vegemite | 19% | Gluten-free bread | 33% |
| Baked^1^ | 24% | Pumpkin | 17% | Eggs | 33% |
| Kumara | 23% | Oats | 17% | Yoghurt – fruit | 33% |
| Rice based cereal | 23% | Fish – non oily | 17% | Tea – herbal | 33% |
| Tea – herbal | 22% | Baked^1^ | 17% | Avocado | 29% |
| Gluten-free bread | 21% | Apple | 15% | Lettuce | 29% |
| Grilled^1^ | 21% | Carrot | 15% | Spinach | 29% |
| Oats | 20% | Rice based cereal | 13% | White bread | 29% |
| Honey | 20% | Tea – herbal | 15% | Cow’s Milk – whole | 29% |
| Marmite or Vegemite | 20% | Grilled^1^ | 15% | Beef | 29% |
| Avocado | 18% | Gluten-free bread | 13% | Honey | 29% |
| Carrot | 18% | Cauliflower | 13% | Grilled^1^ | 29% |

Cooking methods are marked with ^1^.
