# Supplementary material for: Inflammatory Bowel Disease: Are Symptoms and Diet Linked?
Source: Nutrients. 2020 Sep 29;12(10):2975. doi: 10.3390/nu12102975 (PMC7650696; doi:10.3390/nu12102975)
Supplement: Supplementary file 1 [file nutrients-12-02975-s001.zip › Supplementary/nutrients-937697-supplementary edited.docx]

**Supplementary Figure S1.** Inflammatory Bowel Disease (IBD) Questionnaire

Study ID number

______________________

Inflammatory Bowel Disease (IBD)
Questionnaire

| First Name: | | Family Name: | | | | | | | | | | | |
| --- | --- | --- | --- | --- | --- | --- | --- | --- | --- | --- | --- | --- | --- |
|  | | | | | | | | | | | | | |
| **Part 1: General Information and Outdoor Exposure** | | | | | | | | | | | | | |
|  |  | |  |  | | |  |  | | | | | |
| **1.** | What is your gender? | |  | Male | | |  | Female | | | | | |
|  |  | |  |  | | |  |  | | | | | |
|  |  | |  |  | | |  |  | | | | | |
| **2.** | What is your age? | | Years: | | | |  | | | | | | |
|  |  | |  |  | | |  |  | | | | | |
|  |  | |  |  | | |  | | | | | | |
| **3.** | What is your height and weight? | | Height: | |  | cm | |  | ft |  | in |  |  |
|  | *(please list and tick (√) the relevant units)* | | Weight: | |  | kg | |  | lb |  |  |  |  |
|  |  | |  |  | | |  |  | | | | | |
|  |  | |  |  | | |  |  | | | | | |
| **4.** | What is your Ethnicity? | |  | European | | |  | Maori | | | | | |
|  | *NOTE: this information will only be used to describe* | |  | Pacific Peoples | | |  | Asian | | | | | |
|  | *the ethnic makeup of the study population. No* | |  | Middle Eastern | | |  | African | | | | | |
|  | *analysis of this information will take place, nor* | |  | Other (please specify) | | |  |  | | | | | |
|  | *will any specific numbers be disclosed.* | |  |  | | |  |  | | | | | |
|  |  | |  |  | | |  |  | | | | | |
|  |  | |  |  | | |  |  | | | | | |
| **5.** | What form/s of Inflammatory Bowel Disease have | |  | Crohn’s Disease | | |  | Ulcerative Colitis | | | | | |
|  | you been diagnosed with? | |  | Indeterminate Colitis | | |  | Other (please specify) | | | | | |
|  |  | |  |  | | |  |  | | | | | |
|  |  | |  |  | | |  |  | | | | | |
| **6.** | When were you diagnosed with IBD? | | Month: | | | | Year: | | | | | | |
|  |  | |  |  | | |  |  | | | | | |
|  |  | |  |  | | |  |  | | | | | |
| **7.** | Have you experienced active IBD during the previous 12 | |  | Yes | | |  | No (I have remained | | | | | |
|  | months? | |  |  | | |  | in remission) | | | | | |
|  |  | |  |  | | |  |  | | | | | |

Foods or Food Combinations you associate with the **ONSET** of your IBD symptoms
Please tick (√) the food/s you associate with the **onset** of your IBD symptoms (excluding allergies/intolerances that cause discomfort **DISTINCT** from your IBD symptoms) and/or list other foods and/or food combinations below this table.

| **√** | **FRUIT** | **√** | **NUTS / SEEDS /** | **√** | **DAIRY PRODUCTS** | **√** | **SWEETS/SNACKS** |
| --- | --- | --- | --- | --- | --- | --- | --- |
|  | Apple |  | **DRIED FRUIT** |  | Butter |  | Biscuits |
|  | Apricot |  | Almond |  | Cheese – hard |  | Cake |
|  | Avocado |  | Apricot |  | Cheese – soft |  | Chocolate |
|  | Banana |  | Brazil |  | Cream |  | Crackers |
|  | Cherry |  | Cashew |  | Ice-cream |  | Corn chips |
|  | Grapes |  | Cranberry |  | Cow’s Milk – light |  | Dips |
|  | Grapefruit |  | Date |  | Cow’s Milk – whole |  | Lollies |
|  | Kiwifruit |  | Fig |  | Yoghurt – fruit |  | Muesli bars |
|  | Mandarin |  | Hazelnut |  | Yoghurt – dairy |  | Pizza |
|  | Nectarine |  | Macadamia |  | food eg caramel |  | Potato chips |
|  | Orange |  | Peanut |  |  |  |  |
|  | Peach |  | Pine nut | **√** | **MEAT** | **√** | **BEVERAGES** |
|  | Pear |  | Pistachio |  | Beef |  | Alcohol – any |
|  | Pineapple |  | Pumpkin |  | Chicken |  | Beer |
|  | Plum |  | Raisin |  | Fish – non oily |  | Coffee |
|  | Strawberry |  | Sunflower |  | eg hoki, snapper |  | Energy drinks |
|  |  |  | Walnut |  | Fish – oily |  | Fruit juice |
| **√** | **VEGETABLES** |  |  |  | eg salmon, tuna |  | Hot Chocolate |
|  | Asparagus | **√** | **GRAINS** |  | Lamb |  | Milo |
|  | Beans |  | Barley |  | Pork |  | Soft drinks - any |
|  | Beetroot |  | Oats |  | Processed eg |  | Soft drinks – sugar free |
|  | Broccoli |  | Popcorn |  | salami, luncheon |  | Spirits |
|  | Brussels Sprouts |  | Rice |  | Seafood eg mussels |  | Tea – black |
|  | Cabbage |  | Wheat |  | Turkey |  | Tea – herbal |
|  | Carrot |  |  |  | Veal |  | Wine |
|  | Capsicum | **√** | **BREAD** |  |  |  |  |
|  | Cauliflower |  | Brown | **√** | **SAUCES** | **√** | **ADDITIVES** |
|  | Celery |  | Full-grain |  | Barbeque |  | Artificial Sweetener |
|  | Chickpeas |  | Gluten-free |  | Chilli |  | Food colouring |
|  | Chilli |  | White |  | Chutney |  | Herbs |
|  | Corn |  | Wholemeal |  | Mayonnaise |  | Pepper |
|  | Courgette |  |  |  | Maple syrup |  | Salt |
|  | Cucumber | **√** | **CEREALS** |  | Salad dressing |  | Spices |
|  | Garlic |  | Bran based |  | Tomato |  | Sugar |
|  | Kumara |  | Corn based |  |  |  |  |
|  | Leek |  | Rice based | **√** | **SPREADS** | **√** | **COOKING METHODS** |
|  | Lentils |  | Wheat based |  | Honey |  | Baked |
|  | Lettuce |  | Muesli |  | Jam |  | Deep Fried |
|  | Mushroom |  |  |  | Margarine |  | Fried |
|  | Onion | **√** | **MISC** |  | Marmalade |  | Grilled |
|  | Parsnip |  | Eggs |  | Peanut Butter |  |  |
|  | Pumpkin |  | Pastry |  | Marmite or Vegemite |  |  |
|  | Spinach |  | Tobacco |  |  |  |  |
|  | Tomato |  |  |  |  |  |  |
|  |  |  |  |  |  |  |  |
| **Other Foods and/or Food Combinations:** | | | | | | | |
|  | | | | | | | |
|  | | | | | | | |
|  | | | | | | | |

Foods or Food Combinations you associate with the **WORSENING** of your IBD symptoms
Please tick (√) the food/s you associate with the **worsening** of your IBD symptoms (excluding allergies/intolerances that cause discomfort **DISTINCT** from your IBD symptoms) and/or list other foods and/or food combinations below this table.

| **√** | **FRUIT** | **√** | **NUTS / SEEDS /** | **√** | **DAIRY PRODUCTS** | **√** | **SWEETS/SNACKS** |
| --- | --- | --- | --- | --- | --- | --- | --- |
|  | Apple |  | **DRIED FRUIT** |  | Butter |  | Biscuits |
|  | Apricot |  | Almond |  | Cheese – hard |  | Cake |
|  | Avocado |  | Apricot |  | Cheese – soft |  | Chocolate |
|  | Banana |  | Brazil |  | Cream |  | Crackers |
|  | Cherry |  | Cashew |  | Ice-cream |  | Corn chips |
|  | Grapes |  | Cranberry |  | Cow’s Milk – light |  | Dips |
|  | Grapefruit |  | Date |  | Cow’s Milk – whole |  | Lollies |
|  | Kiwifruit |  | Fig |  | Yoghurt – fruit |  | Muesli bars |
|  | Mandarin |  | Hazelnut |  | Yoghurt – dairy |  | Pizza |
|  | Nectarine |  | Macadamia |  | food eg caramel |  | Potato chips |
|  | Orange |  | Peanut |  |  |  |  |
|  | Peach |  | Pine nut | **√** | **MEAT** | **√** | **BEVERAGES** |
|  | Pear |  | Pistachio |  | Beef |  | Alcohol – any |
|  | Pineapple |  | Pumpkin |  | Chicken |  | Beer |
|  | Plum |  | Raisin |  | Fish – non oily |  | Coffee |
|  | Strawberry |  | Sunflower |  | eg hoki, snapper |  | Energy drinks |
|  |  |  | Walnut |  | Fish – oily |  | Fruit juice |
| **√** | **VEGETABLES** |  |  |  | eg salmon, tuna |  | Hot Chocolate |
|  | Asparagus | **√** | **GRAINS** |  | Lamb |  | Milo |
|  | Beans |  | Barley |  | Pork |  | Soft drinks - any |
|  | Beetroot |  | Oats |  | Processed eg |  | Soft drinks – sugar free |
|  | Broccoli |  | Popcorn |  | salami, luncheon |  | Spirits |
|  | Brussels Sprouts |  | Rice |  | Seafood eg mussels |  | Tea – black |
|  | Cabbage |  | Wheat |  | Turkey |  | Tea – herbal |
|  | Carrot |  |  |  | Veal |  | Wine |
|  | Capsicum | **√** | **BREAD** |  |  |  |  |
|  | Cauliflower |  | Brown | **√** | **SAUCES** | **√** | **ADDITIVES** |
|  | Celery |  | Full-grain |  | Barbeque |  | Artificial Sweetener |
|  | Chickpeas |  | Gluten-free |  | Chilli |  | Food colouring |
|  | Chilli |  | White |  | Chutney |  | Herbs |
|  | Corn |  | Wholemeal |  | Mayonnaise |  | Pepper |
|  | Courgette |  |  |  | Maple syrup |  | Salt |
|  | Cucumber | **√** | **CEREALS** |  | Salad dressing |  | Spices |
|  | Garlic |  | Bran based |  | Tomato |  | Sugar |
|  | Kumara |  | Corn based |  |  |  |  |
|  | Leek |  | Rice based | **√** | **SPREADS** | **√** | **COOKING METHODS** |
|  | Lentils |  | Wheat based |  | Honey |  | Baked |
|  | Lettuce |  | Muesli |  | Jam |  | Deep Fried |
|  | Mushroom |  |  |  | Margarine |  | Fried |
|  | Onion | **√** | **MISC** |  | Marmalade |  | Grilled |
|  | Parsnip |  | Eggs |  | Peanut Butter |  |  |
|  | Pumpkin |  | Pastry |  | Marmite or Vegemite |  |  |
|  | Spinach |  | Tobacco |  |  |  |  |
|  | Tomato |  |  |  |  |  |  |
|  |  |  |  |  |  |  |  |
| **Other Foods and/or Food Combinations:** | | | | | | | |
|  | | | | | | | |
|  | | | | | | | |
|  | | | | | | | |

Foods or Food Combinations you associate with **REDUCING** the severity of your IBD symptoms
Please tick (√) the food/s you associate with **reducing** your IBD symptoms and/or list other foods and/or food combinations below this table.

| **√** | **FRUIT** | **√** | **NUTS / SEEDS /** | **√** | **DAIRY PRODUCTS** | **√** | **SWEETS/SNACKS** |
| --- | --- | --- | --- | --- | --- | --- | --- |
|  | Apple |  | **DRIED FRUIT** |  | Butter |  | Biscuits |
|  | Apricot |  | Almond |  | Cheese – hard |  | Cake |
|  | Avocado |  | Apricot |  | Cheese – soft |  | Chocolate |
|  | Banana |  | Brazil |  | Cream |  | Crackers |
|  | Cherry |  | Cashew |  | Ice-cream |  | Corn chips |
|  | Grapes |  | Cranberry |  | Cow’s Milk – light |  | Dips |
|  | Grapefruit |  | Date |  | Cow’s Milk – whole |  | Lollies |
|  | Kiwifruit |  | Fig |  | Yoghurt – fruit |  | Muesli bars |
|  | Mandarin |  | Hazelnut |  | Yoghurt – dairy |  | Pizza |
|  | Nectarine |  | Macadamia |  | food eg caramel |  | Potato chips |
|  | Orange |  | Peanut |  |  |  |  |
|  | Peach |  | Pine nut | **√** | **MEAT** | **√** | **BEVERAGES** |
|  | Pear |  | Pistachio |  | Beef |  | Alcohol – any |
|  | Pineapple |  | Pumpkin |  | Chicken |  | Beer |
|  | Plum |  | Raisin |  | Fish – non oily |  | Coffee |
|  | Strawberry |  | Sunflower |  | eg hoki, snapper |  | Energy drinks |
|  |  |  | Walnut |  | Fish – oily |  | Fruit juice |
| **√** | **VEGETABLES** |  |  |  | eg salmon, tuna |  | Hot Chocolate |
|  | Asparagus | **√** | **GRAINS** |  | Lamb |  | Milo |
|  | Beans |  | Barley |  | Pork |  | Soft drinks - any |
|  | Beetroot |  | Oats |  | Processed eg |  | Soft drinks – sugar free |
|  | Broccoli |  | Popcorn |  | salami, luncheon |  | Spirits |
|  | Brussels Sprouts |  | Rice |  | Seafood eg mussels |  | Tea – black |
|  | Cabbage |  | Wheat |  | Turkey |  | Tea – herbal |
|  | Carrot |  |  |  | Veal |  | Wine |
|  | Capsicum | **√** | **BREAD** |  |  |  |  |
|  | Cauliflower |  | Brown | **√** | **SAUCES** | **√** | **ADDITIVES** |
|  | Celery |  | Full-grain |  | Barbeque |  | Artificial Sweetener |
|  | Chickpeas |  | Gluten-free |  | Chilli |  | Food colouring |
|  | Chilli |  | White |  | Chutney |  | Herbs |
|  | Corn |  | Wholemeal |  | Mayonnaise |  | Pepper |
|  | Courgette |  |  |  | Maple syrup |  | Salt |
|  | Cucumber | **√** | **CEREALS** |  | Salad dressing |  | Spices |
|  | Garlic |  | Bran based |  | Tomato |  | Sugar |
|  | Kumara |  | Corn based |  |  |  |  |
|  | Leek |  | Rice based | **√** | **SPREADS** | **√** | **COOKING METHODS** |
|  | Lentils |  | Wheat based |  | Honey |  | Baked |
|  | Lettuce |  | Muesli |  | Jam |  | Deep Fried |
|  | Mushroom |  |  |  | Margarine |  | Fried |
|  | Onion | **√** | **MISC** |  | Marmalade |  | Grilled |
|  | Parsnip |  | Eggs |  | Peanut Butter |  |  |
|  | Pumpkin |  | Pastry |  | Marmite or Vegemite |  |  |
|  | Spinach |  | Tobacco |  |  |  |  |
|  | Tomato |  |  |  |  |  |  |
|  |  |  |  |  |  |  |  |
| **Other Foods and/or Food Combinations:** | | | | | | | |
|  | | | | | | | |
|  | | | | | | | |
|  | | | | | | | |

**THANK YOU FOR TAKING THE TIME TO COMPLETE THIS QUESTIONNAIRE**

**Extra space** **if required** (please list the question number your answer relates to)

| **QUESTION** | **ANSWER** |
| --- | --- |
|  |  |
|  |  |
|  |  |
|  |  |
|  |  |
|  |  |
|  |  |
|  |  |
|  |  |
|  |  |
|  |  |
|  |  |
|  |  |
|  |  |
|  |  |
|  |  |
|  |  |
|  |  |
|  |  |
|  |  |
|  |  |
|  |  |
|  |  |
|  |  |
|  |  |
|  |  |
|  |  |
|  |  |
|  |  |
|  |  |
|  |  |
|  |  |
